# Cytogenetic and molecular characterization of an oligoasthenozoospermia male carrier of an unbalanced Y;22 translocation: A case report

**DOI:** 10.1097/MD.0000000000015209

**Published:** 2019-04-12

**Authors:** Chunshu Jia, Linlin Li, Shuang Chen, Dejun Li, Xuan Wang, Ruizhi Liu, Hongguo Zhang

**Affiliations:** a Center for Reproductive Medicine and Center for Prenatal Diagnosis, First Hospital; b Jilin Engineering Research Center for Reproductive Medicine and Genetics, Jilin University, Changchun, China.

**Keywords:** genetic counseling, male infertility, oligoasthenozoospermia, Y ;22 translocation

## Abstract

**Rationale:**

Y;autosome translocations are associated with male infertility and azoospermia. Some carriers with a Y:22 translocation can produce offspring and transmit the translocation through generations without phenotypic repercussion. Hence, the clinical features of carriers with certain Y chromosome abnormalities remain uncertain.

**Patient concerns:**

An apparently healthy 33-year-old man, 175 cm tall and weighing 60 kg had a 6-month history of primary infertility.

**Diagnoses:**

The patient was diagnosed with oligoasthenozoospermia. A series of examinations have been performed to evaluate possible genetic causes of this diagnosis. Several methods included semen analysis, hormone measurements, cytogenetic analysis, and high-throughput multiplex ligation-dependent probe amplification semiconductor sequencing.

**Interventions:**

The patient underwent detailed genetic counseling. Cytogenetic analysis was advised for his father. Preimplantation genetic diagnosis was performed to improve potential pregnancy success rate.

**Outcomes:**

Semen analysis revealed oligoasthenozoospermia. Hormone levels were within the normal limits. The karyotype of the patient and his father was 45,X,der(Y;22). Sequencing results indicated the presence of the sex-determining region on the Y chromosome gene. Y-chromosome microdeletion detection showed the presence of AZF (azoospermic factor)a, AZFb, and AZFc regions, but deletion of b2/b3 and duplication of b3/b4 regions.

**Lessons:**

A clinical karyotype report involving a Y chromosome abnormality should consider the results of semen analysis, which helps to identify the chromosomal breakpoint. Semiconductor sequencing technology was useful for clarifying AZF gene microdeletions.

## 1 Introduction

Currently in China, 1 in 4 couples exhibit infertility during their reproductive lifespan.^[1]^ Male infertility is present in approximately 50% of infertile couples,^[2]^ and chromosomal anomalies are found in 10% to 15% of azoospermic men and 7% of all infertile men.^[3]^ Sex chromosome abnormalities are relatively predominant among the karyotypic abnormalities found in azoospermic men.^[4]^ Y;autosome translocations are associated with male infertility and azoospermia.^[5]^ However, some carriers of Y;22 translocations can produce offspring and transmit the chromosomal anomaly through 4 generations without phenotypic repercussions.^[6,7]^ The clinical features of carriers with karyotypes that include Y chromosome abnormalities remain uncertain.

We describe an oligoasthenozoospermic male with an unbalanced translocation 45,X,der(Y;22) inherited from his father. This investigation used cytogenetic and semiconductor sequencing methods.

## 2 Methods

This study was approved by the Ethics Committee of the First Hospital of Jilin University (No. 2018-373) and informed written consent was obtained from the patient for publication of this case report.

### 2.1 Semen analysis and detection of reproductive hormones

Semen analysis was performed in accordance with World Health Organization (WHO) standard protocol.^[8]^ Serum follicle-stimulating hormone (FSH), luteinizing hormone (LH), prolactin (PRL), estradiol (E_2_), and testosterone (T) were determined by using a commercially available kit and the Elecsys 2010 chemistry analyzer (Roche, Germany).

### 2.2 Cytogenetic analysis

Extracted from peripheral blood of the patient, lymphocytes were cultured for 72 hours in RPMI-1640 medium. Conventional metaphase chromosomal karyotype analysis was performed from peripheral blood lymphocytes using G-band staining according to standard cytogenetic procedures.

### 2.3 Molecular analysis

Human blood samples were collected from the patient. High-throughput multiplex ligation-dependent probe amplification semiconductor sequencing was performed using genomic DNA from peripheral blood. Semiconductor sequencing was performed according to our previously study.^[9]^ Markers included 36 sequence-tagged sites, as follows: AZF (azoospermic factor)a region (sY84, sY86, sY81, sY85, sY182, sY608, sY741, sY1323, sY2323), AZFb region (sY127, sY134, sY121, sY124, sY128, sY130, sY133, sY117, sY850, sY1002, sY2597, sY2832, sY2833), and AZFc region(sY254, sY255, sY145, sY152, sY153, sY157, sY239, sY242, sY802, sY856, sY1191, sY2713, sY2900, sY2928), with sex-determining region on the Y chromosome (SRY) and zinc finger protein, X-linked/zinc finger protein, Y-linked used as internal controls.

## 3 Case description

**Figure 1:**
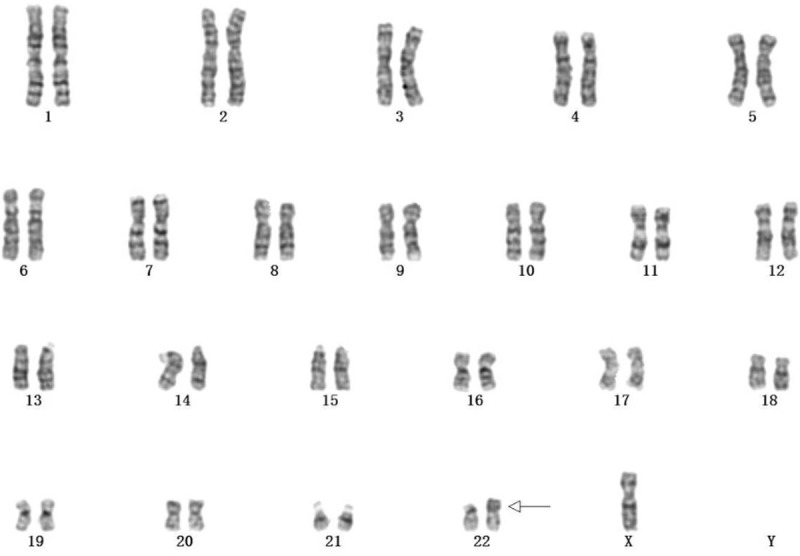
Karyotype of the case was found to include chromosome 45,X,der(Y;22).

An apparently healthy 33-year-old man had a 6-month history of primary infertility. He exhibited a well-developed male phenotype and was 175 cm tall and weighed 60 kg. Physical examination showed a normal male habitus except for slightly smaller testes. Scrotal echography showed the left and right testes were approximately 10 and 12 mL in volume, respectively. Repeated semen analysis revealed oligoasthenozoospermia. Hormone analysis showed FSH, LH, E_2_, serum PRL, and T levels were within the normal limits. Cytogenetic analysis showed that the patient had unbalanced Y-22 chromosome translocations, although the exact position of breakpoints was unclear. With informed consent, the patient's parents refused to undergo cytogenetic investigations. Male patients suggested the presence of the *SRY* gene. Hence, we assumed that the karyotype was 45,X,der(Y;22) (Fig. 1). After genetic counseling, karyotype analysis showed his father was 45,X,der(Y;22). We further examined AZF microdeletions. Clinical features of this patient included oligoasthenozoospermia, which suggested the presence of the Y chromosome AZF gene. The sequencing results showed the presence of *SRY*. Y-chromosome microdeletion analysis showed the presence of AZFa, AZFb, and AZFc regions, and the deletion of b2/b3 and duplication of b3/b4 regions. After genetic counseling and informed consent, this patient will seek assisted reproductive technology treatment combined with preimplantation genetic diagnosis.

## 4 Discussion

Clinical phenotypes of Y:autosome translocations are dependent on the precise translocation breakpoints on the Y chromosome.^[10]^ The *SRY* gene is responsible for testicular determination, and is located on the short arm of the Y chromosome.^[11]^ It is considered to be a master gene regulating the cascade of testicular determination,^[12]^ and is critical for initiating testis development of the bipotential gonad and differentiation of Sertoli cells. Sertoli cells play important roles in the differentiation and development of the male germline.^[12]^ Deletion in the SRY region of human Y chromosome involving t(Y;22) resulted in a female phenotype.^[13]^ In present study, our case showed normal hormone levels and male phenotype, except for slightly smaller testicular size, suggesting the Yp region is not missing. From the karyotype shown in Figure 1, we speculated that the short arm of the Yp region combined with the short arm of chromosome 22.

The AZF, located on Yq11, is essential for spermatogenesis and may be affected secondary to a microdeletion or complete loss resulting from translocation.^[14]^ It is generally assumed that sterile males with Y:autosome translocations have a Y chromosome breakpoint at Yq11, which contains the AZF locus.^[15]^ The clinical phenotypes of Y:autosome translocations are also related the Y chromosome microdeletions.^[16]^ In general, carriers of AZF-deletions on the long arm of the Y chromosome appear to be azoospermic.^[17]^ In this study, the male displayed oligoasthenozoospermia. The semiconductor sequencing showed the presence of AZFa, AZFb, and AZFc regions. Combined with the karyotype of the patient's father, we speculated that the karyotype of the patient was 45,X,psu dic(Y;22). This carrier is likely to have retained fertility because the Y chromosome still included the SRY and all AZF regions, consistent with previous literature reports of 45,X,psu dic(Y;22) carriers.^[6,7]^

The AZF region of the Y chromosome is classically divided into the AZFa, AZFb, and AZFc regions. The AZFc is the most vulnerable to deletions, as it contains repeated sequences and palindromes.^[18]^ Complete AZFc deletions are associated with severe oligozoospermia.^[19]^ In addition, the AZFc locus may have some partial deletions, including b1/b3 (1.6 Mb), b2/b3 (1.8 Mb) and gr/gr (1.6 Mb).^[20]^ Our case exhibited b2/b3 deletion and b3/b4 duplication. However, the correlation of b2/b3 deletion and male infertility was poor, and ethnicity dependent, and there was no significant difference in the frequency of prevalence of b2/b3 deletions in fertile and infertile Asian men.^[18,21]^ Further research is needed to confirm the correlation of b3/b4 duplication and oligoasthenozoospermia.

A limitation of this study is the lack of detailed research regarding the specific molecular effect by molecular-cytogenetic methods. Therefore, we are unable to explain the relationship between this translocation and spermatogenesis.

In conclusion, this study reports a case of oligoasthenozoospermia in a patient that inherited a chromosome Y;22 translocation from his father. A clinical karyotype report involving a Y chromosome abnormality should consider the results of semen analysis, which helps to identify the chromosomal breakpoint. Semiconductor sequencing technology was useful for clarifying potential involvement of AZF gene microdeletions.

## Acknowledgments

Authors thank Charles Allan, PhD, from Liwen Bianji, Edanz Editing China (www.liwenbianji.cn/ac), for editing the English text of a draft of this manuscript.

## Author contributions

**Funding acquisition:** Ruizhi Liu.

**Investigation:** Linlin Li, Shuang Chen.

**Methodology:** Dejun Li, Xuan Wang.

**Writing – original draft:** Chunshu Jia.

**Writing – review & editing:** Ruizhi Liu, Hongguo Zhang.

Hongguo Zhang orcid: 0000-0001-8953-863X.
